# Exploring Tinnitus-Induced Disablement by Persistent Frustration in Aging Individuals: A Grounded Theory Study

**DOI:** 10.3389/fnagi.2017.00272

**Published:** 2017-08-10

**Authors:** Nicolas Dauman, Soly I. Erlandsson, Dolorès Albarracin, René Dauman

**Affiliations:** ^1^CAPS-EA4050, Department of Psychology, University of Poitiers Poitiers, France; ^2^Department of Social and Behavioural Studies, University West Trollhättan, Sweden; ^3^INCIA, UMR Centre Nationnal de la Recherche Scientifique, University of Bordeaux Bordeaux, France

**Keywords:** tinnitus, frustration, disablement, long-term suffering, intra-individual variability, grounded theory, qualitative research

## Abstract

**Background:** Qualitative research can help to improve the management of patients, meet their expectations and assist physicians in alleviating their suffering. The perception of moment-to-moment variability in tinnitus annoyance is an emerging field of exploration. This study sought to enlighten variability in tinnitus-induced disablement using a qualitative approach.

**Methods:** Twelve participants (six females, six males, aged 51–79) were recruited via the French Tinnitus Association Journal for participation in recorded semi-structured interviews. Each participant had three interviews lasting 1 h, the sessions being separated one from the other by 2 weeks. Following recommendations of Charmaz (2014), the second and third interviews were aimed at gathering rich data, by enhancing the participants' reflexivity in the circumstances of distress caused by tinnitus. After transcription, the data (*n* = 36 interviews) were analyzed using the approach to Grounded Theory proposed by Strauss and Corbin (1998).

**Results:** Tinnitus as persistent frustration emerged as being the core category uniting all the other categories of the study. Hence, the core category accounted for the broader scope in participants' experience of chronic tinnitus. It is suggested that tinnitus-induced disablement varied according to the degree of frustration felt by the participants in not being able to achieve their goals. The implications of this were analyzed using the following categories: “Losing body ownership,” “Lacking perspectives,” and “Persevering through difficulties.” Based on these findings, we draw a substantive theory of tinnitus tolerance that promotes an active, disciplined and individualized approach to tinnitus-induced disablement. The model distinguishes pathways from sustained suffering to reduced annoyance (i.e., emerging tolerance). It accounts for difficulties that the participants experienced with a perceived unchanged annoyance over time. Furthermore, this model identifies a set of new attitudes toward oneself and others that tinnitus tolerance would entail.

**Conclusion:** The subjective experience of frustration enlightens tinnitus-induced disablement, offering new perspectives for long-term self-management. Modulation of frustration, rather than moderation of tinnitus interference, is suggested as a new approach to the clinical management of tinnitus-related distress.

## Introduction

Disablement is a concept that refers to the impact of a chronic condition on “people's abilities to act in a common, expected and personally desired way in society” (Verbrugge and Jette, 1994, p. 3). Understanding the disablement process not only accounts for the disabilities that an individual might meet as a consequence of his or her illness; it can in a broader sense also explain the impaired interactions that occur in the individuals' social and physical environment. This process involves a close interest for reciprocal influences between the disabled individuals and others' attitudes toward those restrictions. From a psychosocial perspective, chronic illness was characterized as a threat to self-integrity and “taken-for-granted assumptions about possessing a smoothly functioning body” (Charmaz, 1995, p. 657). It is accompanied by the loss of bodily enjoyment and inevitable limitations in the exercise of one's own desire. Charmaz (1995) further emphasized that chronically suffering patients may undertake divergent relationships toward their illness, whether they refuse the weakening of their life (i.e., they struggle against the condition) or adapt their way of living to accommodate to physical losses. Adaptation to a chronic condition is not a linear process. When body limitations and suffering exceed the threshold of tolerability, frustration may become overwhelming for the disabled individual (Dow et al., 2012).

Over the last decades, numerous studies have documented disabilities reported by tinnitus patients. Heterogeneity of outcomes in the tinnitus literature was recently stressed as a methodological issue that should be considered in a broader, ecologically valid framework (Searchfield, 2014). Recent studies have documented complex changes in patients' perception of their sound environment after tinnitus onset. Enhanced salience of tinnitus in either silent or noisy environments was reported by Pan et al. (2015). The authors point to a diversity of needs among tinnitus patients, reporting that perceived loudness in a noisy place could contribute to worsening of tinnitus in 32% of their population (*n* = 258). Hébert et al. (2013) also demonstrated that a tinnitus patient group (*n* = 124) displayed an increased growth in loudness compared to a control group with only hearing loss (*n* = 106). Matching the level of hearing loss in compared groups, they further suggested that hypersensitivity to noise in tinnitus patients was a phenomenon distinct from loudness recruitment. While tinnitus and hyperacusis do not always overlap clinically, the findings by Hébert et al. (ibid) support the relevance of identifying a sub-group of patients for whom specific treatments may be beneficial (Dauman and Bouscau-Faure, 2005; Schecklmann et al., 2014). Vielsmeier and co-authors reported recently that more than 70% of patients with tinnitus (*n* = 351) experienced difficulties understanding speech (Vielsmeier et al., 2016). While these difficulties were a concern for 40% of the patients when no specific circumstance was considered, 80% acknowledged that speech understanding was a problem in a cocktail party situation, with multiple sources of noise.

Beyond changes in the relationship to the sound environment, studies have documented extra-auditory difficulties among patients with tinnitus. Tinnitus has been considered to be the main sleep deprivation factor among people with hearing impairment (Test et al., 2011). Apart from difficulties falling asleep, tinnitus manifests as the reason for early awakening and mid-sleep awaking, which often lead to the use of hypnotic medication (ibid). Schecklmann et al. (2015) reported that the prevalence of insomnia in a group of chronic tinnitus patients (*n* = 182) reached 76%. Otherwise, lack of sleep can substantially contribute to increase tinnitus-induced disablement. According to Pan et al. (2015) up to 27% of their sample (*n* = 258) reported that lack of sleep was a source of increased annoyance. Tinnitus interference with mental performances has also been documented in the literature (for a review see Mohamad et al., 2016). It was suggested that the level of demand of tasks (Stevens et al., 2007) and executive control deficit (Heeren et al., 2014) contribute to patients' impairment when conflicting information is to be sought during a task. The consequences of tinnitus on social relationships are far less documented, although it was found that relatives' misunderstanding of daily difficulties accompanied by tinnitus could be a source of frustration for patients (Tyler and Baker, 1983; Sullivan et al., 1994).

Aspects of variability in how patients cope with their condition could be found in a treatment trial including 37 patients with severe refractory tinnitus (Zöger et al., 2008). The authors explored the effects of a group psychotherapy approach in comparison with anti-depressive medication. For example, some patients in the group reported in a post-therapy interview that tinnitus varied with their emotional state. Those who felt that they had gained a deeper personal insight into how their suffering from tinnitus varied were also those who were likely to perceive a positive long-term effect as a result of the therapy. These results suggest that patients' narratives could be used in the search for intra-individual variability of tinnitus based on patients' histories. Previous studies of the psychoacoustics of tinnitus have shown to what extent tinnitus may vary (Penner et al., 1981). Burns (1984) confirmed the high intra-individual variability of results in terms of equalization of pitch, intensity, and narrowband noise levels required to continue to mask the tinnitus. Emphasis was placed on perceptual differences between tinnitus and external sounds, the latter having a seemingly much higher stability. Another example is to be found in a magnetoencephalographic study (MEG) carried out some 15 years ago (Dietrich et al., 2001) with eight subjects with tinnitus who presented a dip in hearing on high frequencies. The study demonstrated broader MEG responses when the sounds used to stimulate the auditory system were situated in the lesion-edge frequency region compared to responses obtained with sounds from pre-lesional frequencies (corresponding to audiometrically healthy zones). One of the subjects was tested four times in 3 months. Quite surprisingly, his MEG responses were not at all stable between the tests. The overrepresentation of lesion-edge frequencies disappeared in a few weeks but reappeared in the next session, before showing up in the last session at a level even higher than in the first one (Figure 4. of Dietrich et al. (2001) article). The authors showed that the observed MEG variations had no link with the perceived seriousness of tinnitus as measured by the Structured Tinnitus Interview (Goebel and Hiller, 2001).

Investigations conducted in a large series of patients also provided information on tinnitus variability. In a sample of 528 tinnitus patients, Stouffer and Tyler (1990) reported the following: (i) only half (52%) considered their tinnitus pitch as stable, and more than a third (36%) perceived fluctuations in pitch during the day; (ii) tinnitus intensity was considered as stable in only 44% of the cases; changes in intensity could occur suddenly (25%) or progressively (31%); (iii) since tinnitus onset annoyance was mainly considered as stable (60%), but 34% of the sample reported more intense tinnitus, and only 7% a lessening of its severity. The study also revealed several factors likely to increase tinnitus, such as staying in a quiet place, being exposed to a lot of noise, and being subjected to stress and lack of sleep. Interestingly, an investigation conducted by Slater et al. (1987) in nearly 1,000 individuals living in Wales mentioned the relief frequently felt by patients when they were engrossed, involved or interested enough in an active occupation. On the other hand, most of the patients had a hard time at night and needed to take sleeping pills or tranquillizers to get to sleep. These findings suggest that individuals may modulate their awareness and annoyance of tinnitus through their behavior (Roberts et al., 2013). However, such an influence may be limited considering the course of tinnitus annoyance in many patients. A previous study from our group (Bouscau-Faure et al., 2003) confirmed the variability factors mentioned in other studies, but these proved elusive to translate into a global handicap score. Data collected from 57 patients at the Clinique des Acouphènes (Tinnitus Clinic, University Hospital) were subjected to a detailed analysis of individual answers to the Iowa questionnaire THQ (Tinnitus Handicap Questionnaire, Kuk et al., 1990). For each item, subjects were invited to answer with a score between 0 and 100, and could note down a free-form comment in another box corresponding to the question. Importantly, to two precise questions: “I am unable to relax because of tinnitus” (item 14) and “I have trouble falling asleep at night because of tinnitus” (item 16), it was not uncommon for subjects to write “It depends on my level of nervousness and tiredness; I hesitate between 50 and 100%”. Emerging ecological momentary assessment (EMA) is advocated to overcome these difficulties inherent to one-time static measurements, thereby enabling more accurate measures of moment-to-moment tinnitus variability (Wilson et al., 2015). Preliminary studies suggest that intra-individual variability may be much higher in tinnitus than traditionally thought. A remaining challenge of EMA in tinnitus is that participants are not always able to account for the variability of annoyance that they encounter (Schlee et al., 2016). To date, variability in tinnitus has mainly been investigated by using self-rating questionnaires to score the impact of tinnitus on patients' daily life (e.g., Kuk et al., 1990; Zeman et al., 2012) and to assess treatment outcomes (e.g., Newman et al., 2014). Questionnaires are usually based on personality constructs (e.g., anxiety, depression, acceptance, self-efficacy) that are *applied to* the experience of tinnitus (e.g., Smith and Fagelson, 2011; Weise et al., 2013). However, such measures may not be sufficient for specific features of the experienced condition to emerge. An alternative methodology, like Grounded Theory, uses unstructured data (i.e., individual interviews) and a constant coding procedure to discover constructs that are close to the data. This inductive approach goes beyond the pre-structured instrument level and explores variations in difficulties experienced by participants on an individual level.

In the current study, we propose to contribute to the understanding of the variability of tinnitus-induced disablement from such a qualitative approach, by investigating the inner perspective of a small group of participants who have lived with bothersome tinnitus for some time. Qualitative inquiry and analysis have been promoted by Malterud (2001) with the aim of improving management strategies and better understanding the clinical setting, patients' expectations and physicians' attitudes toward the suffering of their patients. In audiology, qualitative research has previously underlined the contribution of the patients' perspective on the management of hearing impairments (Hallberg and Carlsson, 1993; Laplante-Lêvesque et al., 2012), Méniere's disorder and otosclerosis (Eriksson-Mangold et al., 1996; Erlandsson et al., 1996), hearing aid use and non-use in older people (Lockey et al., 2010), and young people's risk-taking as regards exposure to loud music (Widén and Erlandsson, 2007). To our knowledge, however, no qualitative study has yet been undertaken in tinnitus, especially with regard to the issue of intra-individual variability of tinnitus-induced disablement, and how this relates to tinnitus suffering and to tolerance. From previous studies (Dauman and Erlandsson, 2012; Dauman et al., 2015) we hypothesized that tinnitus patients' narratives offer a substantial contribution to the comprehension of the condition, by taking into account the perspective of individuals in the perception of the circumstances in which their tinnitus varies.

### Aims

This study had three aims. First, we wanted to use a more systematic approach (i.e., qualitative research) to investigate tinnitus-induced disablement. Secondly, we aimed at contextualizing the suffering of tinnitus in a social perspective, i.e., taking into account ecological validity by exploring the participants' narratives. The objective was to allow individuals to play an active role in data collection, and acknowledging their agency in how meaning is constructed with regard to their condition. Thirdly, we aimed to enlighten their attitudes toward health professionals in order to increase knowledge about the difficulties they may encounter in their search for a cure.

## Materials and methods

### Study design

This qualitative study was based on Grounded Theory, a well-known research methodology within the field of health and chronic illness (Glaser, 1978; Strauss and Corbin, 1998; Charmaz, 2014). Our approach was especially influenced by Strauss and Corbin's (1998) mode of Grounded Theory data analysis (described below) and by Charmaz's (2014, 1995) approach to open-ended interviews that promotes rich data collection through sensitized listening. The aim was to deepen our understanding of distress and disablement in individuals with chronic tinnitus, through a sequential and intensive interviewing of participants. To achieve this goal, we designed a study consisting of three interview sessions for each participant, each interview lasting 1 h with an interval of 2 weeks between. It was considered that this design would contribute to the building of trust between the participants and the researcher (ND). The second and the third interviews with each participant provided detailed data, with a contribution of the researcher's reflexivity emerging from memo-writing and initial coding between each interview. However, it is acknowledged that this first study using Grounded Theory encountered limitations with respect to theoretical sampling. We address this issue at the end of the article and draw some perspectives for future studies.

### Ethical considerations

The Ethical Research Committee CPP Sud-Ouest Outre Mer III (DC2016/154) approved the study. Each participant was handed an information letter stating the purpose of the study. Prior to participation they were assured that the study would be anonymous and that any personal information would remain confidential. They were told that in order to enable a rigorous analysis of their discourse, the interviews had to be recorded and that the study results would be published in a scientific journal. All of them acknowledged that it was important to contribute to research on chronic tinnitus and gave their written informed consent in accordance with the Declaration of Helsinki (WMA, 2013).

### Procedure

The recruitment of participants was conducted with the help of the French Tinnitus Patients Association (France Acouphène, FA). A call for testimonials under the title: “How does one live with tinnitus nowadays?” was published by FA. The national recruitment aimed at exploring a variety of participants' health paths, depending on their residence (i.e., metropolis, medium-sized cities, villages). Individual interviews were conducted via Skype or by phone. By selecting members of the Tinnitus Patients Association to participate in our investigation, we expected to receive data rich enough for a qualitative methodology like Grounded Theory. The participants had had a rather long experience of living with persistent tinnitus and were also used to sharing their experiences with other members of the association. In this way, we believed that their reflections and thoughts would be highly valuable for the investigation and also suit the study methodology.

### Participants and demographics

Table 1 shows the 12 participants by gender, in ascending order of hearing loss. Their median age was 63 years and the majority (9/12) were no longer working for health reasons. At the time of the interviews the median duration of tinnitus was 14 years (range 6–37 years). Seven participants considered that the discomfort due to tinnitus had worsened after the initial onset, while others reported stabilization or waning of symptoms over time. A minority reported more difficulties in falling asleep than before the onset (3/12). However, a majority perceived tinnitus as soon as they awakened with difficulties getting back to sleep (10/12). Finally, only two subjects used hearing aids regularly. The sample was gender-balanced.

**Table 1:** Study participants by gender and in ascending order of hearing loss, with information on tinnitus perception, overall annoyance, sleep, and wearing of hearing aid.

**Sex**	**Age**	**Uni HL**	**Bi HL**	**AA00 HL**	**HF HL**	**Tinnitus lateralization**	**Tinnitus duration**	**Type**	**Predominant description**	**Overall annoyance**	**Insomnia before**	**Insomnia after**	**Drugs**	**Tinnitus at awaking**	**Hearing aid use**
**F1**	75		X	20	55	Left	13 y	Noise	Humming	Reduced	No	No	No^*^	Yes	No
**F2**	79		X	25	60	Left	6 y	Noise	Whistling, horn	Enhanced	Yes	Yes	Yes	Yes	No
**F3**	67		X	30	40	Left	8 y	Noise	Crackling, roaring	Enhanced	No	No	No^*^	Yes	No
**F4**	60	X		38,5	42.5	Head	11 y	Noise	Rumbling	Unchanged	No	Yes	No	Yes	No
**F5**	63	X		73,5	75	Left	13 y	Noise	Crackling	Reduced	No	No	No	No	Yes (unilat and occasional)
**F6**	56		X	120 46,5	120 47.5	Left	29 y	Noise	Roaring, whistling	Unchanged	No	Yes	No	Yes	Yes (bilat and regular)
**M1**	56		X	15	42.5	Left	12 y	Noise	Whistling	Enhanced	No	No	No^*^	Yes	No
**M2**	51		X	21,5	50	Both	8 y	Noise	Whistling, crackling	Enhanced	No	Yes	Yes	Yes	No
**M3**	61		X	26,5	67.5	Head	13 y	Sound	Whistling	Enhanced	Yes	Yes	Yes	Yes	No
**M4**	64		X	31,5	72.5	Left	17 y	Sound	Whistling	Enhanced	No	No	No	Yes	No
**M5**	57	X		35	112.5 Prog	Right	32 y	Sound	Crackling	Enhanced	No	No	No	No	No
**M6**	62		X	40	120 Prog	Right	16 y	Sound	Whistling	Unchanged	No	No	No	Yes	Yes (bilat and regular)

*Legend. For each sex group, the ranking of participants based on the amount of hearing loss was determined by averaging the pure-tone hearing thresholds at 500, 1,000, and 2,000 Hz of the poorer ear, according to Merluzzi and Hinchcliffe, 1973. In addition, the average hearing loss over the high frequencies, 4,000 and 8,000 Hz, was calculated in the same ear. By comparing the two hearing threshold averages, it can be seen that hearing loss predominated over the high-frequencies in the majority of participants*.

* *means that participants only used a drug when they needed to be efficient the next day*.

### Rich data gathering

The interviews began with an interview guide (see Table 2) based on previous studies on patients' narratives (Dauman and Erlandsson, 2012; Dauman et al., 2015). Five open-ended questions were retained as an outline for the semi-structured interviews, aimed at helping participants to contextualize their experience of tinnitus i.e., to explore circumstances preceding the onset of tinnitus and possible long-term physical and mental health consequences. Information collected included duration, description and laterality of tinnitus, discomfort since onset of symptoms, tinnitus perception (sound/noise), use of hearing aid and the impact of tinnitus on sleep. Other questions focused on the participants' close networks such as family and entourage and strategies undertaken in order to manage suffering and on help received from healthcare providers.

**Table 2:** List of initial questions used in first interview with participants.

1. Can you tell me how your tinnitus began?
2. What steps have you taken since then?
3. To this day, what are the consequences of tinnitus on your family and social life?
4. What do you do to lessen your suffering?
5. What do you think of the therapeutic approaches provided?

While conducting the interviews, the first author used memos to record initial codes, e.g., participants reactions and experiences (e.g., being overwhelmed, hypersensitivity) and associated ideas and questions about circumstances that arose during the interviews. More abstract memos were used to collect and cluster events of the same type into a broader description of tinnitus in daily living. Significant themes were identified (e.g., sensitivity to noise, tiredness, being misunderstood, suffering), which allowed the second and third interviews to include more focused questions on tinnitus disablement (e.g., What do you do when you have to deal with new worsening of your suffering?; Have you encountered disbelief from others about its seriousness?). Focused questions were asked as a means of enriching descriptions of difficulties when this proved necessary. More abstract memos enabled us (ND & SE) to build the first concepts that clustered and highlighted seemingly different conducts by a suggested underlying process. For instance, comparison between avoiding noises and surrounding oneself with pleasant sounds led to the idea that controlling sounds was an important feature of participants' relationship with their environment. Hence, an initial theme “sensitivity to noise” contributed to an important concept, later labeled “auditory congruence”. In turn, abstract memos helped us to formulate more precise questions (e.g., What do you need to feel comfortable?). Box 1 shows an excerpt of abstract memos regarding the need to control one's surrounding auditory environment, leading to focused questions with a more precise content.

Box 1 **Excerpt from abstract memo about the concept “Controling auditory environment.”**

Having tinnitus all day long prone to control one's surrounding environment. Circumstances in which too much noise are to be endured must be avoided (e.g., restaurant, bar, cinema) because of the consecutive worsening of tinnitus. Sudden, unpredictable noises from the outside world seem to bring the worst annoyance (e.g., fire engine, moped). Noise pollution is also a problem when people are talking all together. Decline any invitation may be the only solution, when you don't know if you could participate in meetings. Hard days cannot be foreseen with chronic tinnitus. Then, even sounds from relatives may be too much to endure. Withdrawal can be mandatory for avoiding worsening the situation. Instead of other's sounds, it seems more desirable to surround oneself with sounds that are pleasant and choosen (e.g., music, fontain, radio). These ones may bring comfort, helping to relax a bit from tinnitus presence. They ensure that there can be less discomfortable situations, from time to time. Surrounding oneself with these sounds is even more necessary at the tinnitus onset.

### Extended data analysis

The material collected consisted of 36 in-depth interviews (*n* = 12 participants; 3 interviews per participant). In total, the transcripts from the recordings ran to 678 pages. The detailed material allowed for more systematic scrutinizing of data, which led to the emergence of a central perspective (i.e., a core category) with broad implications for the data material (Glaser, 1978). The core category was judged to be the best fit and to explain variations in the data. Data transcription enabled a constant comparison between codes and concepts, which were easier to cluster and contrast based on the coding procedure. According to the recommendations of Strauss and Corbin (1998), we followed a three-step procedure which was carried out by ND and discussed at each stage with the co-authors (SE, DA, RD) who offered remarks and significant comments. Table 3 explains the overview of the coding process, including data gathering and extended analysis. The material was first subjected to an open coding with systematic reading of the transcripts, line by line. Our understanding of the participants' daily life situation was significantly improved by this initial stage of analysis. It helped to sustain a balance between previous concepts from data gathering and emergent ones (i.e., involvement, sustainability, independence). DA contributed with reflections regarding the outcomes of open coding and helped in building logical links between individual behaviors. Abstract memos enabled a personal “dialogue” with the data, by the construction of a storyline based on the concepts. This led to performing a second axial coding that combined concepts about similar individual behaviors and circumstances. The rationale of using axial coding was to further increase conceptual density around as few axes as possible. This step entailed searching for reciprocal implications between categories (e.g., articulate “facing disbelief from others” with “developing independence toward misunderstanding”). SE and RD established the appropriateness of the categories and sought the best fit with clinical issues arising from the analysis. Reflexive questions were addressed to foster understanding of axis of analysis with respect to their conditions and consequences (e.g., Why do meaningful activities help in sustaining tolerance? Why is it essential to regulate one's lifestyle?). From conceptual tensions around axes of analysis, a more central perspective was found to account for each axis of analysis and a governing principle between them could be drawn. The last step of selective coding consisted in reconsidering all the results using the perspective that the core category provided. This led SE and ND to search for literature review that would best fit their tentative labeling, and to find more suitable terms to identify the core category and supporting categories. The core category was assumed to offer a high level of explanation of the participants' experience (Glaser and Strauss, 1967; Glaser, 1978) by integrating variations across the participants (Strauss, 1987).

**Table 3:**
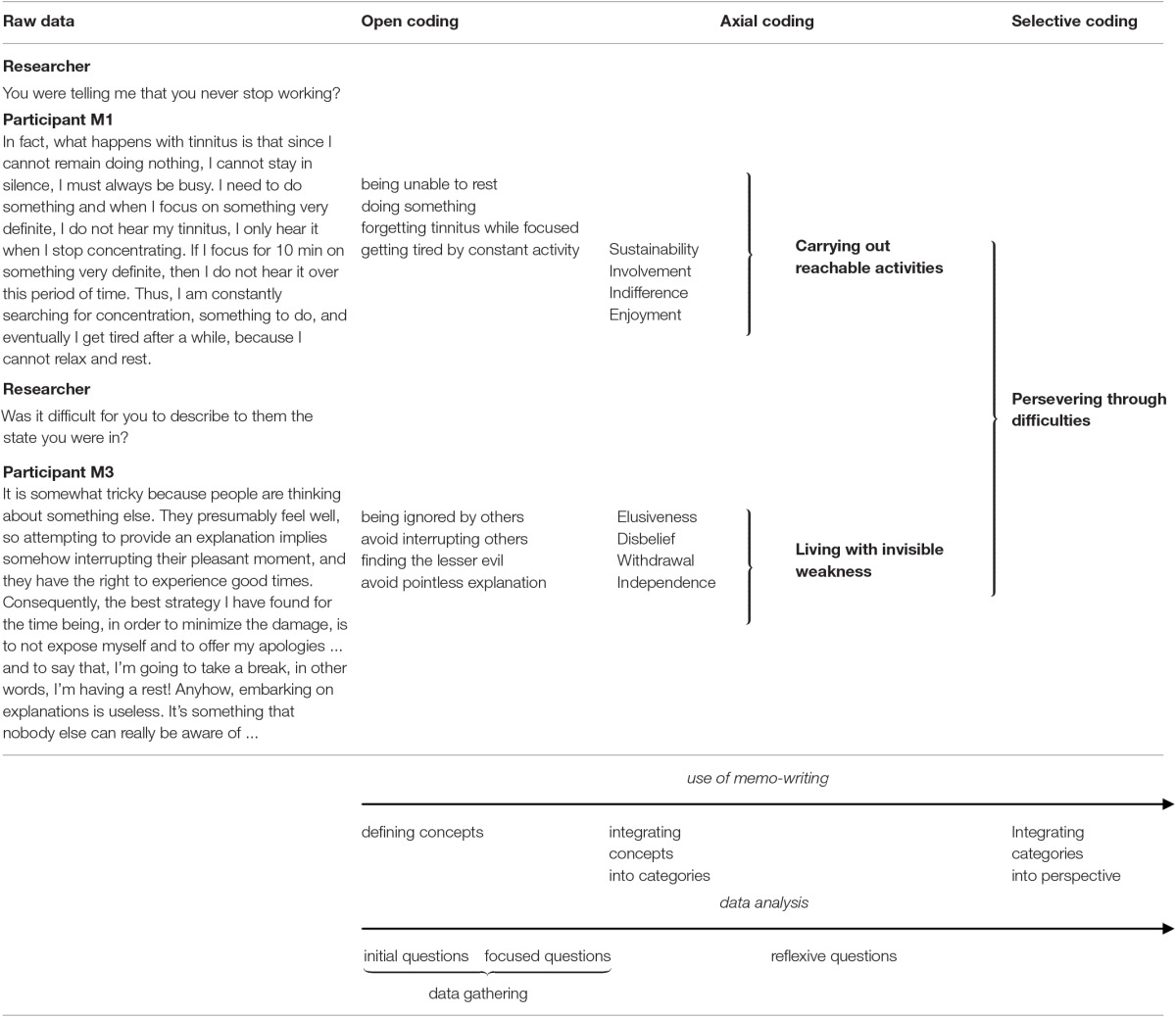
Flowchart of the coding process in relationship to data gathering and data analysis.

## Results

### Tinnitus as persistent frustration

Tinnitus was found to vary in relation to the participants' frustration at being unable to achieve their goals. The term “frustration” is used throughout the results to indicate the subjective experience of being unable to change a situation or to fulfill one's desire. Even though the participants' frustration was mainly related to the auditory field (e.g., having difficulties to communicate in noisy environments), it also concerned other circumstances in which hearing does not play a central role (e.g., when others misunderstand the impairment caused by tinnitus). We hypothesize that variability in tinnitus annoyance reflects participants' inability to act in accordance with their desire in the experiences they have of the condition. In particular, the participants' perceptual awareness of their inability to change the situation seemed to be important to them. The more acute their awareness of the situation was, the greater was the tinnitus annoyance in their perceptual field. Although all sufferers reported variability in their annoyance with tinnitus, their frustration was not easy to grasp, as the following remark illustrates:

“I cannot compare it with anything else, I don't know, it ruins my life, it prevents me from doing things I'd like to…it bothers me whatever I try to do.” Participant F6.

We identified three main implications of tinnitus as a persistent source of frustration: 1. Losing body ownership, 2. Lacking perspectives and 3. Persevering through difficulties. Table 4 presents an overview of the results.

**Table 4:** An overview of results emerging from the Grounded Theory approach to data.

**TINNITUS AS PERSISTENT FRUSTRATION**		
**1. Losing body ownership**	**2. Lacking perspectives**	**3. Persevering through difficulties**
1.1. Being invaded by inescapable noise	2.1. Failing to identify tinnitus	3.1. Living with invisible weakness
1.2. Holding on to a fragile body	2.2. Facing an irreversible condition	3.2. Carrying out achievable activities

## Losing body ownership

Tinnitus was found to interfere with the fulfillment of the participants' daily goals. It impinges upon their free will and body enjoyment, interfering with the perception of sounds and making communication difficult in noisy environments. It deprives them of the calming experience of silence. It leads them to undertake activities constantly in order to distract themselves from a phenomenon that above all they want to get rid of. Participants try to counterbalance this unpleasantness by creating an enjoyable sound environment. They also try to regulate their lifestyle in order to limit the variability of their tinnitus and its interference in their lives. However, this task is relentless because of the unyielding symptoms of the condition.

### Being invaded by inescapable noise

Tinnitus is perceived as a disagreeable noise. Its intensity varied from one moment to the other, sometimes with extreme awareness of its presence at the heart of the participants' perception. The consciousness of the obstacles they meet when trying to control their sound environment even makes tinnitus worse. It interferes with the search for informative sounds, and with the desire to escape noises that interfered with concentration and rest. In all cases, when they are thwarted in achieving respite, participants report a worsening of their tinnitus that increased and prolonged their frustration. The relationship between variability of tinnitus and experienced frustration varied with the individual's ability to control one's sound environment. The most hearing-impaired participants reported that difficulties in perceiving sounds of low-to-medium intensity (e.g., a fan, fridge or coffee-maker) made their tinnitus even more bothersome.

“I think that from the moment I focus on a sound that I like to hear clearly and there is a background noise, my brain is disturbed and the tinnitus increases, or at least my perception of tinnitus increases […] I feel as if the more my ears are prepared to hear, the more likely that my tinnitus does the same and increases.” Participant M6

A resolute but unsuccessful effort to listen to sounds could also intensify the presence of tinnitus in the perceptual field. This can happen when someone must pay attention to important information in a professional framework, or needs to focus on a sound of uncertain origin. Searching for sounds that they were unable to grasp could be experienced as a worsening of tinnitus (somewhat similar to the experience in a soundproof chamber, an environment which is known to temporarily enhance the perception of tinnitus).

“The worst is the room where we took the audiogram. When they told me, behind the window, to: ‘Listen to the sound’, I felt burning everywhere and then, after a while, a sound appeared besides the tinnitus that I was supposed to hear…the tinnitus was even stronger, maybe because I was expecting this sound I was supposed to hear, I don't know…” Participant F6.

Hearing loss increases the effort made to understand what others are talking about. It is a source of fatigue accumulated during the day, because of the greater attention required to listen and answer appropriately. It forces the hearing-impaired to ask others, whose reply is not always comprehensible, repeat, and this has a negative influence on the conversation. Meeting difficulties in communication situations, the hearing-impaired have to deal with a more intense form of tinnitus that seems to compete with their intention to participate in conversations.

“At first, it was as if I was wearing a helmet, as if there was something around my head that isolated me completely…and if I wanted to hear when someone talked to me, I had to pull out my ear, take it out of the helmet.” Participant F5.

The hypothesized relationship between frustration and tinnitus annoyance is also shown in the participants' thwarted desire to protect themselves from noisy environments. In their experience, the onset of tinnitus heralded a lower tolerance threshold to noise disturbance. Following the onset of tinnitus, sudden noises (e.g., a fire siren) and noises lasting in time (e.g., festivities in the street) are perceived as harmful to the integrity of the body. The reluctance to be exposed to an uncertain sound environment is also dependent on the increased intensity of tinnitus, which can last from one to a few days. The awareness of such a disturbance is perceived intensely during exposure to noise, when being forced to endure passively a constraint that cannot be avoided. In such circumstances sufferers must go on, against their will, to the limits of their resistance to noise disturbance.

“I know immediately when the evening is going to be too noisy. And when I depend on others to accompany me home, it is really annoying…Because I know that it means that my tinnitus will be back for several days. And that my ears will be buzzing if I expose myself to the noise for too long […] I then huddle up over my discomfort and irritation and the pleasant moments are gone. I count the minutes go by while thinking: ‘When is it going to end?’” Participant F1.

The insistence of tinnitus, from waking up in the morning to difficulties falling asleep, reinforces the sense of its constant presence in one's mind. Some of the participants are preoccupied by this continual appearance of tinnitus with very rare moments when they are able to pay less attention to the sound. Most of them are also confronted with the presence of tinnitus when they wake up at night. Such experiences give the impression that tinnitus is a phenomenon that is omnipresent in their perception.

“The problem is that you have to make a constant effort. Just to live. You need to make an effort whatever you do, in order to sleep, for everything. It is not natural to make an effort all the time, that's what wears the body out. […] Tinnitus consumes life, it is so oppressive and intrusive that it steals a part of your life.” Participant F4.

This conviction is not contradicted by any perception of a cessation of tinnitus. In anguish, individuals may project their incapacity to put up with tinnitus on their future existence. The absence of the prospect of improvement leaves them with a deep feeling of helplessness.

“I wrote some notes that I used to put in my pockets. They said ‘Yes, I accept to live with it’. Because at first, I couldn't tell myself ‘You're going to put up with this all the time, all your life’. It is not possible. […] And then, once the day was over, even if it had been a bad day, I wrote on the paper ‘Yes, well done’ or ‘To improve’. And when I was in a lot of pain, I put my hand in the pocket and took out the paper to remember what I had to do.” Participant M3.

The unyielding nature of tinnitus can push individuals to the limits of their physical endurance. Some cut themselves off from social relations, only seeking to protect themselves from noise pollution which seems to have a negative influence on tinnitus. Above all, they want the agony to cease, so that they can return to the life they used to have. Faced with the absence of silence, some evoke their fear of going mad. For others, the lack of silence is experienced as their biggest frustration. Not being able to perceive silence again is regarded as a fundamental loss in the experience of oneself in the absence of others. If there is no solution to this kind of suffering, tinnitus can at times become overwhelming.

“I am invaded by noise. When I listen to the television, people speak too loud. Family meals are no longer possible, because of loud sounds when there are too many people around…I no longer celebrate Christmas with my family, I don't feel capable of managing all this, I prefer to isolate myself rather than being invaded by the noise, it's unbearable.” Participant M4.

### Holding on to a fragile body

Confronted with the interference of tinnitus with their hearing experience, participants reported trying to restore the balance between their desires and their sound environment. They adapt the latter to their auditory sensitivity, using recordings (e.g., fountain, natural music) or radio chats to exercise efficiently the desire to choose sounds that bring comfort. Such individualized environments enable them to listen to enjoyable sounds that counterbalance the unpleasantness of tinnitus. Sounds that are personally selected are of special value, also because they are easily accessible.

“I listen to music for pleasure; I like to listen to music. Well, tinnitus is still in the background, but I enjoy the music, so I don't really care about the tinnitus.” Participant F5.

The use of auditory protection in a noisy professional environment fulfills the same purpose with respect to the avoidance of noise disturbance. It enables sufferers to contain the gap experienced between noise intensity that they can tolerate and the noises they are exposed to, being actors in settings they cannot escape.

“The first thing I think about when I go to work is to put in my earplugs. I couldn't do it without my earplugs. I never forget them. If I do, I get worried, stressed. […] But I use different types of filters, depending on if I am at work, outside or at home.” Participant M2.

Of great importance is to choose how to deal with the sound environment. It is experienced as the assurance of having the choice to select and overlook sounds that are to be perceived.

“My wife keeps telling me: ‘I don't understand why you have that around your neck and why you don't use headphones’. But no, headphones are not convenient for me, because the sounds I hear don't interest me all the time. […] I don't know how to say it, it is an area of interest for me that I can take on board or drop whenever I want.” Participant M6.

Regulation of lifestyle was a second adjustment made by the participants. It implies a stricter management of rest and sleep compared to how routines used to be before the onset of tinnitus. Participants stressed the importance of rest and sleep, as well as the need to sometimes isolate themselves despite the frustrations it might give rise to socially. Lifestyle regulation also includes avoiding any excess of noise, agitation and sometimes stimulants (e.g., alcohol, coffee). In particular, lifestyle regulation enables them to limit the variability of tinnitus-induced disablement and avoid crises resulting from going over their limits.

“The two main factors are fatigue and noise exposure. If I handle them well, the variability of tinnitus becomes less difficult to handle. But if I fail to handle it, I experience huge variability and the tinnitus can be dreadful.” Participant M1.

Trying to cope with the frustration over enduring, permanent tinnitus may lead some individuals to adopt sleeping on their own. This is an attempt to avoid fighting against tinnitus, and instead admitting to its presence in their mind and body.

“At night, I still prefer to manage my tinnitus in silence and complete darkness. Going to sleep is complicated… So, I cut myself off from external noise, and come to terms with it: ‘here you go, you hear it…’ I know that at other moments during the day, I won't hear tinnitus for some time. That's what I try to tell myself.” Participant M4.

Sufferers often reported limiting their socializing in order to alleviate their frustration being exposed to others' noisy behavior and misunderstandings of what the hearing loss implies. Simultaneously, they escape from potentially demanding situations, which also increase tinnitus annoyance. Even though all sufferers regretted it, these social limitations seem to be efficient in a long-term perspective. Nevertheless, the negative side is that the strategy restricts relationships with close family and friends.

“Before, we were a family who organized many meals together. But now the social part of my life changed a lot. For some time, I had almost no social contacts; now I'm beginning again, slowly…organizing meals with 6–8 persons. Afterwards I need two, three days to recover, as my ears are more sensitive, and there is systematically a worsening of my tinnitus.” Participant M2.

Considering that nothing alters the sustainability of tinnitus leaves many participants with a sense of helplessness. It is described as an inescapable struggle against it.

“…You end up completely empty at night, knocked out. You think: ‘Yet I didn't do much during the day’ Yes! You were hiding, fighting against this parasite that is here permanently, 24 h a day, you were fighting to try and do something else. […] you fight, but after a while, you don't have any energy left to overcome the parasite…” Participant M5.

Even for those who are able to develop some occasional or even more regular tolerance to its presence, invasive thoughts about tinnitus remain a potential risk. Constant effort is needed in order to avoid paying attention to it and at the same time holding on to a controlled lifestyle. If not, tinnitus can “get the upper hand” over the desire not to be dominated by it. Tinnitus is perceived as a handicap to be carried within oneself, whereas the most natural reaction would be to get rid of it once and for all.

## Lacking perspectives

That something strange is happening in the body becomes an urgent worry after tinnitus onset and is something most participants have experienced. This belief feeds their need to understand what is taking place in their brain and ears, hoping to find a means to act upon it. These expectations are rarely a matter of concern for physicians who view tinnitus as a symptom of brain activity and not as an illness. Perceived as an unreachable goal, the prediction that by time you can get used to it accentuates their sense of incapacity to put up with it. Furthermore, a chat with their physicians regarding the daily experience of tinnitus would allow them to understand more fully how to manage tinnitus-induced disablement. With no perspective of understanding the variability of tinnitus and its influence on daily life, patients are left with their frustration to manage.

### Failing to identify tinnitus

Tinnitus presence may elicit some serious questioning about the origin of noise in the body which continues for years after tinnitus onset, as shown by a large number of medical consultations. Patients are determined not to miss any solution of how to get rid of it and want to explore every potential source (e.g., ear, brain, teeth, cervical vertebra, etc.). The belief that tinnitus reveals an underlying pathology triggers the search for medical explanations. In a long-term perspective, tinnitus onset may induce preoccupations and thoughts about a degradation of hearing and normal brain activity. Concerns about managing tinnitus without therapeutic help is strengthened by the lack of a satisfactory medical response to the possible etiology. Without a clear understanding of a likely cause and the process of tinnitus, no efficient means of action seem to be at hand.

“When it happens to you, you ask yourself: ‘Why me? Why is this happening to me?…while I always had a quiet life, I paid attention to what I ate, what I did. What on earth have I done to deserve this?…It's human, I think, to ask oneself these questions. And we have no answer.” Participant F2.

Medical explanations often alleviate preoccupations about the danger of tinnitus for hearing and brain activity. However, its variability over time also prompts questions like: “what is happening in my brain?” Being able to understand more about tinnitus variability might have helped the participants to better cope with the suffering. This kind of knowledge, however, is seldom addressed in medical consultations. The lack of medical explanation regarding this aspect of tinnitus makes patients observe themselves through the prism of their own physical experience.

“At the time, I even did Excel tables on my blood pressure, the intensity of my emotions, and tinnitus intensity. I gathered information, as if I was a witness as well as a patient, to somehow be able to understand what was going on!” Participant M3.

Participants highlight the role of internal factors (e.g., tiredness, anxiety) as well as external circumstances (e.g., noises, upsetting events) that might worsen tinnitus-induced disablement. Sometimes, however, tinnitus variability is hard to understand. It may increase when they are engaged in things they have to do, and may compete with their activities, as if tinnitus is “trying” to oppose what they like to do. Some witness a kind of automatic rolling-out of bodily changes, against which they cannot fight.

“It fluctuates. Today it is bearable, but yesterday it wasn't. Tomorrow it will return more strongly…It is really strange. It changes all the time. One day it is strong, the next it is less, so you never know…But I know the day it will be intense. Today it is not virulent, but tomorrow it will be, as if to make me pay for the day when it was somewhat quieter.” Participant F2.

### Facing an irreversible condition

Participants' belief that they need to be cured from tinnitus does not fit with their physicians' perspective on their health status. Referring to the absence of a visible pathology on MRI scans, physicians try to reassure and clear up the patients' concerns about it. However, the absence of an underlying pathology does not resolve the suffering. It rather means that nothing can be done for what some sufferers experience as agony. Unintentionally, this kind of information may even worsen the patient's view on his or her experience of tinnitus.

“It is not all about having audiograms, there should be some intervention about our suffering. […] Doctors don't seem to take us seriously, it makes me angry. Once we tell them we have tinnitus, they raise their arms, as if to say there is nothing they can do…” Participant F2.

The examinations that they received (e.g., routine hearing loss measurement) were quite different from the help and care that they felt they really needed. Their practitioners' view about the condition counteracts their belief that “something must be done” to relieve them from the agony that tinnitus causes. Invariably, participants remember words like: “there is nothing to be done against tinnitus” and they “should better get used” to a noise they will certainly hear “all their life”. According to them, practitioners may not realize the impact that such words have on a distressed patient. Indeed, after tinnitus onset most of them did not consider any other solution than relief or suppression of it through treatment.

“At first, you feel really overwhelmed by it. So you think you will remain this way all your life. First because the ENT specialist I met told me so. He said: ‘Your hair cells are destroyed, they will not grow again. So, you will have tinnitus all your life’. […] When a physician tells you that, it's crazy…” Participant F5.

Furthermore, participants missed having a conversation with their physicians regarding how they experienced and reacted to the onset of tinnitus. Little or no information on the process of habituation to the symptom was reported. The way physicians explained the habituation process seemed unfathomable to the sufferers, and was even perceived by some as a source of resignation. There were no questions on how they managed the variability of tinnitus that could have helped them to clarify their search for suitable coping behavior. A perceived reluctance from professionals to talk about how tinnitus might fluctuate from one day to another reinforced participants' feeling that they were receiving no medical help. The perceived closed-mindedness of the medical attitude regarding the characteristics of tinnitus might complicate the early stages of their experience.

“At first, they told me several times: ‘don't worry Ms., you'll get used to it, it's not serious, it will get better. You are going to live well with it—anyway, you are going to live with it–so you better begin right away.’ When I only wanted one thing: to get rid of it. I didn't want to hear this. I couldn't hear it. […] They were perfectly right, ultimately, but in my opinion, they were wrong to be right at once. What they said disturbed me as much as the tinnitus.” Participant F1.

## Persevering through difficulties

The experience of being disturbed by tinnitus cannot be shared and often remains unknown to others. Disclosing the hearing impairment is met with disbelief or a lack of regard from interlocutors, which adds to the participants' feeling that they are suffering from an unrecognizable condition. Therefore, they have to find ways on their own to alleviate the hardship related to the impairment. All of them observed variations in the course of their tinnitus-induced disablement. Some of them realized that these variations gave them the opportunity to change their attitude toward it. For example, activities that they are involved in might enable them occasionally to pay less attention to it. These coping experiences are rewarding and full of meaning because they can restore the congruence between what participants want to do and are able to do, enabling them to live momentarily with less suffering.

### Living with invisible weakness

The invisible presence of tinnitus puts the participants in an awkward position when it comes to social relations. Most of the time, tinnitus is non-existent in contexts of social communication. When informed about it, interlocutors usually appear indifferent or disconcerted. Even close relatives are uncertain about how to react, and hence the subject might create relational tensions. Participants generally decide not to mention tinnitus, even when they are with acquaintances and family members who usually soon forget all about it. To those who do not have their own experience of tinnitus it sounds elusive.

“People who do not have tinnitus don't really manage to grasp the fact that it is permanent. I have friends and family who regularly ask me: ‘Do you still hear it, now?’ Yet, I told them several times that it is permanent, but I don't know…It is a reaction as permanent as tinnitus is, I think.” Participant F5.

Not mentioning tinnitus enables those who suffer to avoid negative comments from people in their entourage. It is aggravating to receive comments about the supposed banality of tinnitus from people who believe it is a lesser problem.

“When I first had the symptoms, people used to tell me: ‘you listen to yourself too much, don't listen to yourself, etc. You're getting upset for nothing, I had tinnitus too, etc.’ I quickly understood that I couldn't talk about it too much, because I'm not understood. People see me as someone who exaggerates, as a hypochondriac. […] I only really talk about it when I am very, very tired, and feel crestfallen.” Participant M1.

To unwillingly choose social isolation enable the participants to limit their frustration (for oneself and others) stemming from people' misunderstanding of their lesser endurance to loud noise. Participating in social gatherings is bearable if it results from a clear and assumed decision. When the noise gets louder, it becomes mandatory to leave the group and rest for a while. The company of others must not prevail over the pressing need to protect oneself from harmful noises. This requires an independence of mind with regard to others, to communicate a trouble that is not shared, and likely to arouse comments. There is a need sometimes to impose limits on the behavior of young children, and to remind others not to speak all at once. Long-term tinnitus management demands living in accordance with one's individual needs more than ever before, and to stick to these new limits.

“I have one day, two days of activity and usually after two days tinnitus is very loud, because I can't put up with it anymore. […] Now I know myself, but before I became depressed, because I went too far…Now I try to slow down, to take breaks. I withdraw more often; I don't get in contact with people so often anymore, whereas before I used to wear myself out.” Participant F4.

### Carrying out achievable activities

All participants notice some kind of reconnection with tinnitus and an increase in its loudness after they have finished an activity they were involved in. Some are surprised to find how easily it comes back into their consciousness, and directly becomes more audible. They would like to know about the mechanisms that make it return as soon as they stop what they are doing.

“Manual work is the most efficient: even in silence. I manage not to hear it then. When I am very busy manually, that is good, I am settled down, my work is perfect and then I don't hear it. But once I stop and look at what I did, it returns. Once I stop concentrating, it returns as fast and goes crescendo, it slowly increases and after 5 min, it's at full pitch.” Participant M1.

This feature of tinnitus can drive some participants to maintain a high level of activity. It is also a way to avoid passive suffering from it. Nevertheless, over time, active opposition toward tinnitus proves not to be efficient. An acute awareness of such limitation reduces the perceived relevance of these activities, which may appear insufficient to alleviate the presence of tinnitus. Unlike sustained efforts to overcome it, someone just mentioning tinnitus can immediately recall its presence to a sufferer who was not paying attention to it just before. Likewise, upon the cessation of a musical activity, tinnitus emerges as strong as ever. Also, a worsening of tinnitus follows a sudden noise.

“Sometimes I hang out with friends, we talk for an hour, and everything's fine. And then, all it takes is that someone tells me: ‘It seems your tinnitus is getting better, no?’ only that, and I answer: ‘Yes, it's somewhat better, it depends on the time’, but then, only with that, I feel tinnitus buzzing in my head. Just talking about it puts it back into my brain.” Participant M6.

For some participants, the experience of the contrasting variations of tinnitus can shed a new light on the phenomenon. Those who realize that they need to restrict their agenda no longer constantly engage in activities. They understand that their thoughts were absorbed in their hobby the moment before tinnitus suddenly became more intense. For such participants, the brief disappearance of tinnitus is experienced as more valuable than its sudden return into their consciousness.

“One day, I realized that I didn't hear it for a while. Then, I found it fantastic. It meant that I managed not to have it in my head anymore! […] But I think it's because I heard it again that I realized I wasn't hearing it before. I don't know in which order it went but…you think about it, you hear it, there is no mystery.” Participant F5.

They rediscover the meaning of being positively involved in one's hobbies. This makes them realize that their attempt to escape from tinnitus is more effective when they cease to distract themselves from it. When those who better tolerate it are absorbed in meaningful activities, they are not concerned by its presence or absence; instead they are positively indifferent toward its variability and presence. Listening to music for the pleasure it gives is different from listening to music with the aim at limiting the disablement of tinnitus. The renewed exercise of one's desire detached from tinnitus allows some participants to get a new perspective. It means that they worry less about its virtual presence when sleeping or not paying attention to it. The belief that an absence of impairment can be achieved through the free exercise of one's will testifies to the pointlessness of fighting against its presence. Thus, activities that participants have learnt to engage in become pleasant and gratifying. They are easy to access and are adapted to their mental and physical preferences. Lasting for some time, they might enable them not to be continuously interrupted by monitoring their tinnitus. In particular, such gratifying activities are associated with the perceived interests of others. For example, an interesting conversation has a positive influence on participants' attention (and thereby on the presence of tinnitus) whereas a boring conversation has no effect at all on it.

“In this self-help association, I found people listening to me in a way the medical profession doesn't. So, my reaction was to return what I received. I thought, if I cannot fight against tinnitus, instead of being passive, I intend to fight for it, within the association. […] We speak with people who experience the same thing, we help them, and it enables us to put things into perspective.” Participant F3.

The fulfillment of pleasant activities convinces some participants that it is possible to escape from tinnitus, without wearing oneself out in a systematic and unsuccessful opposition to it. For example, the pleasure of swimming offers greater comfort than that of permanently listening to the radio in order not to hear it. Spending hours doing craftwork in a workshop relaxes participants more than constantly keeping the mind occupied to forget tinnitus. Writing in the silence of the night is more rewarding than music in the background that is supposed to diminish its presence. The awareness of this possibility offers a wider perspective, greater hope as well as energy for managing tinnitus despite its inevitable variations. Although, it does not guarantee complete control of tinnitus on a day-to day basis, it might lead to greater confidence in the future.

## Discussion

### The relevance of using the term frustration in chronic tinnitus

It has been recently advocated that an ecological framework should integrate the diversity of tinnitus outcomes into an overall construct of tinnitus experience (Searchfield, 2014). The individual who perceives tinnitus is assumed to be at the core of such an ecological framework. Results of the present study suggest that frustration may enlighten this individual perspective. Frustration as a concept (Berkowitz, 1989) fulfills two important criteria to account for tinnitus annoyance, namely variability and ecological validity. Variability of frustration has been demonstrated in experimental settings (Yu et al., 2014). Asked to complete a rewarded task on a computer, individuals who were thwarted in their attempts experienced greater frustration as their motivations increased. Consistent with the frustration-aggression model (Berkowitz, 1989), subjects' frustration instigated immediate aggressive trends, with higher probability to show open aggression with greater prior expectations. Therefore, frustration as a dynamic construct would be suitable in further investigations of tinnitus variability. The ecological validity of frustration has been documented by studies in chronic pain (Schneider et al., 2012), accounting for a broader scope of patients' difficulties than depression, anxiety or fear (Wade et al., 1990). Frustration in patients with pain can be used as a measure of the perceived lack of understanding from others with regard to the strain that they experience in social contexts (Dow et al., 2012). This testifies to the disruptive consequence of pain on one's existence, impacting life roles and the ability to keep perspectives on one's future (Harris et al., 2003). Finally, frustration addresses patients' perception of medical diagnosis and management, which has been previously underestimated in the field of pain (Dow et al., 2012). We believe that the frustration hypothesis in chronic tinnitus may also contribute to a broader understanding of tinnitus distress, incorporating patients' impairments with expectancies about tinnitus management.

### Modulating frustration: a substantive theory of tinnitus tolerance

**Figure 1:**
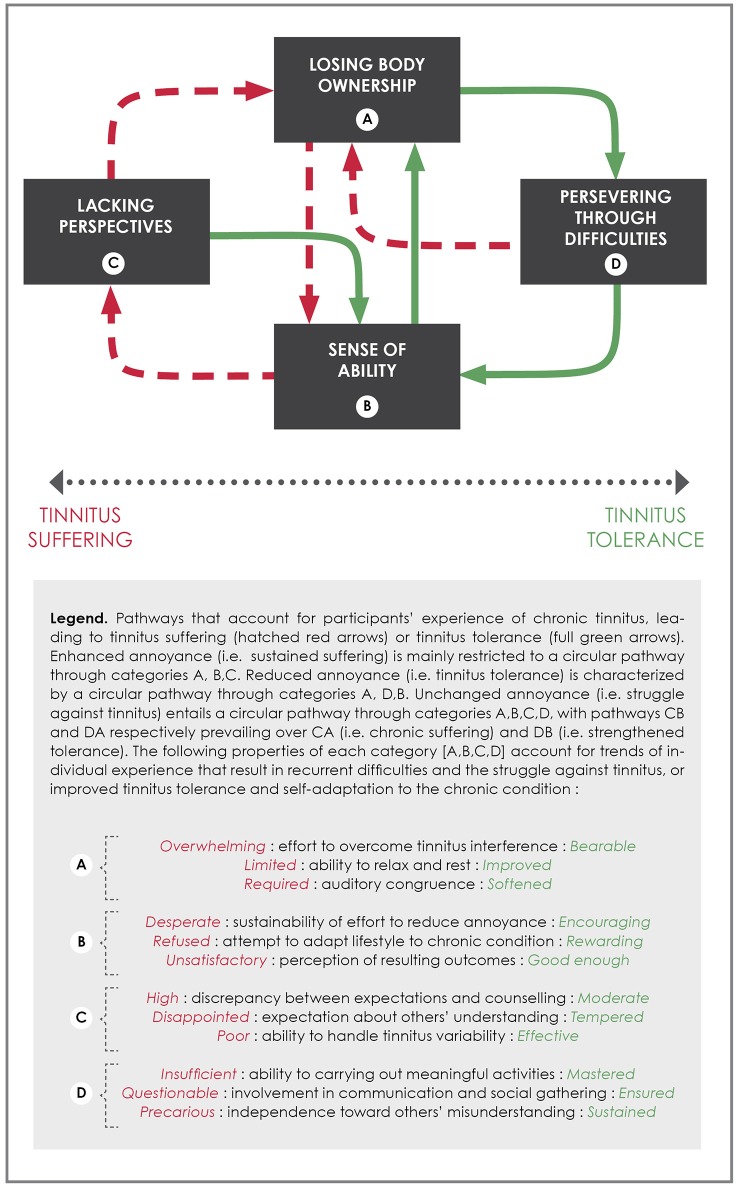
Modulating frustration: a substantive theory of tinnitus tolerance.

In the following section, the analysis of the frustration hypothesis in tinnitus-induced disablement is extended with the building of a substantive theory of tinnitus tolerance. A substantive theory consists in exploring relationships between categories that were presented in the results section (see Table 4). It accounts for trends in individual experience and changes over time, from one experience to another. It further aims at accounting for which difficulties participants meet and how they try to overcome them (Glaser and Strauss, 1967). Eventually, a substantive theory allows hypotheses to be tested in further research. The following figure (Figure 1) establishes predictable relationships between trends in individual conduct in a consistent model that is suitable for implementation in clinical practice. It includes the three previous categories from the results section, “Losing body ownership”, “Lacking perspectives” and “Persevering through difficulties”. A fourth category, “Sense of ability”, is introduced to account for changes in the individual experience of tinnitus, from tinnitus suffering (pathways in hatched red arrows) to tinnitus tolerance (pathways in full green arrows). The figure is based on a continuum of experience that is represented by the horizontal two-way arrow, whose ends lead to suffering and tolerance. A basic assumption of the model is that tolerance to tinnitus entails a sustained and disciplined effort that is challenged by the many circumstances of daily living. In contrast with current models of tinnitus annoyance, this assumption suggests that tolerance cannot be equated with a passive process (e.g., the extinction of a conditioned reflex or over-reaction toward tinnitus stimulus, for a review see Dauman et al., 2013). Therefore, we suggest that the term *tolerance* better describes an active effort from the individual not to suffer from tinnitus than the term habituation. Tinnitus tolerance entails a full reform of oneself with regard to restriction and adaptation of activities, as much as to perceived outcomes that can be achieved through daily efforts.

*Losing body ownership* sets the scene for the understanding of tinnitus-induced disablement and its relationship to persistent frustration. The model predicts that the more effort the individual has to make to overcome tinnitus interference with daily activities, the weaker he or she will feel facing tinnitus in the long term (pathway AB). Difficulties in resting and being able to relax, along with a strict and rigid control over the sound environment (i.e., a required auditory congruence), increase the burden of accepting tinnitus in the perceptual field. This results in a poor *sense of ability* and a lack of sustainability of effort that is required to tolerate its presence. The model further predicts that an affliction like this does not empower the individual to adapt his or her of lifestyle to the condition, but rather to refuse the mere perspective of dealing with tinnitus in the future. Adaptation seems initially unreachable to somebody who is severely disabled by the condition. The frustration hypothesis enables health professionals to consider that such an experience of tinnitus calls for a radical solution to the condition (i.e., tinnitus suppression or significant alleviation) and will not be satisfied with any other moderate perspective (i.e., improving one's tolerance to the condition over time). A poor sense of ability is also prone to undervaluing emerging experiences of lesser annoyance when involved in achievable activities that seem disproportionate to the overwhelming suffering of each and every moment. The model predicts that discrepancy between individual's expectations and counseling contributes to the *lack of perspectives* for the disabled individual (pathway BC). A large number of consultations represent the desperate search for another perspective than merely having tinnitus for the rest of one's life. In addition to disappointment concerning the perceived attitude of professionals (i.e., a lack of concern for one's disablement), the misunderstanding of one's entourage about the new restrictions stemming from tinnitus add to the frustration and the feeling of not being helped by anyone. The model predicts that frustration is worsened by the lack of such perspectives on tinnitus, which results in a vicious cycle leading back to *losing body ownership*. In the figure, sustained suffering is restricted to this cycle A, B, C, with no external factor that might enable the individual to break out of his or her perceived disablement induced by tinnitus.

If individual expectations toward professional counseling are to remain high, the search for external help can strengthen a poor sense of ability to deal with tinnitus on one's own. This paradox is raised by the frustration hypothesis, all the more in that the individual has to learn to live with tinnitus as a chronic condition. Therefore, the role of health professionals must not be undervalued when restoring a sense of ability in disabled patients. To begin with, confident and trustful professionals can help suffering patients to find a new perspective regarding their condition. Setting up a dialogue concerning the condition may help the patient to adhere to the variability of annoyance, and to identify moments and circumstances during which a lesser presence of tinnitus is experienced (when absorbed in achievable activities). Increased insight regarding the experience of tinnitus may support efforts to reduce the annoyance of tinnitus, thus setting a perspective in which potential improvements could come about following expert advice. Another way to reduce the discrepancy between expectations and counseling is illustrated by participants who suddenly cease to consult health professionals and thus, re-focus their efforts to hold on to their body ownership. The model predicts that highly thwarted expectations prevent any improvement in the sense of ability, while moderation might contribute to lesser tinnitus interference. This entails adapting one's lifestyle to the chronic condition (pathway BA), in contrast to the previous struggle against its presence. Regulating one's socializing and fostering one's endurance are strategies that enable the individual to develop ways to put up with the presence of constant tinnitus. An improved ability to rest and a softened auditory congruence (emerging self-confidence toward the sound environment) come by changing one's attitudes and lifestyle, i.e., identified as *persevering through difficulties* (pathway AD).

From this new pathway, a set of outcomes is predicted to emerge in the individual's experience of tinnitus. The most important is assumed to be carrying out meaningful and pleasant activities which are easily achievable and enable the individual to detach her or himself from the interference of tinnitus. Involvement in communication and social gathering might also bring new insights that diminish the presence of tinnitus. Interesting conversations and captivating interactions can be a distraction that eases the experience of tinnitus. *Perceived outcomes* from these new attitudes toward tinnitus interference are assumed to play a central role in distinguishing reduced annoyance (i.e., improved tolerance, pathway DB) from unchanged annoyance (i.e., merely struggling to limit tinnitus annoyance, pathway DA). In the latter, meaningful and pleasant activities appear to be insufficient and disproportionate to the amount of effort that is required by the unyielding presence of tinnitus. Likewise, communication and interaction offer only restricted periods of relief and are questionable when one contrasts them with the virtual omnipresence of tinnitus. When individuals find that it comes back easily into their consciousness, the effort they have to make in order to cope with it seems somewhat trivial. Moreover, they cannot see that these long-term strategies to escape tinnitus can be successful. Prolonged and unchanged annoyance is dealt with by a struggle to limit its presence through engagement in distracting activities, and is to be distinguished from improved tolerance implying self-induced relief from tinnitus. The model predicts that distracting activities have a limited influence upon tinnitus annoyance over time. It further assumes that the effort to overcome tinnitus interference might again be overwhelming sooner or later for patients who struggle against its presence (regressive pathway DA). Difficulties are predicted to reoccur if perceived outcomes lack in sustainability. It is assumed that difficulties in putting up with tinnitus might be limited by previous experience, as shown in the figure by pathway CB (tempered expectations toward others' help and self-confidence in finding relief) rather than CA (sustained suffering with no perspectives over the condition). Therefore, unchanged and prolonged annoyance is characterized by a circular pathway between the four categories A, B, C, D with pathways CB and DA prevailing over sustained suffering (CA) and improved tolerance (DB).

An improved tolerance is characterized by a distinct attitude toward tinnitus (pathways DB). The model predicts that a *sustained and positive* (i.e., not monitored) involvement in meaningful activities can strengthen the sense of effectiveness in distracting oneself from tinnitus interference. This pathway contrasts with previous attempts to merely cover up its presence in the perceptual field. It is assumed that sustainability of effort should be encouraged by the extension of time resulting in less annoyance from it. This new effectiveness of lifestyle is rewarded by the fresh perspective of being able not to suffer *every* moment of life despite its presence. Finding that they are not continuously interrupted by tinnitus monitoring can make individuals realize that they were *not* thinking about it when they were involved in enjoyable activities. According to the model, retraining oneself with respect to its presence might also change the individual's perceived efforts to integrate the condition into her or his lifestyle. The experience of tolerance can confirm one's ability to limit tinnitus interference (pathway BA) and to enjoy pleasant and uninterrupted activities (pathway DB). The model predicts that a virtuous cycle like this (A, D, B) might improve the individual's ability to be satisfied, and also to allow him to be capable of considering future outcomes as *good enough*. Thus, the model does not equate tinnitus tolerance with an achieved stage of perception, but rather defines it as an improved management of the frustration that it causes.

Current psychological therapies for patients with tinnitus attempt to allow sufferers to cope with the implications of tinnitus frustration. *Lacking perspective* may be the primary concern for Cognitive-Behavioral Therapy which has, for decades, emphasized the need to structure patients' cognitions so that they can appraise their condition. Current hypotheses, which highlight the importance of belief in the tinnitus experience, suggest that advances in CBT will take this search for the ability to cope one step further (see McKenna et al., 2014). Following the trend for approaches raising awareness of mindfulness, more recent psychological approaches to tinnitus address the issue of *losing body ownership*. Mindfulness-Based Stress Reduction Therapy (MBSRT, see e.g., Roland et al., 2015) and Acceptance and Commitment Therapy (ACT, see e.g., Westin et al., 2011) have both led to improvement in tinnitus tolerance. Basically, mindfulness promotes the development of a non-judgemental and non-reactive attitude toward the present-moment self-experience (Zou et al., 2016). It entails self-training exercises in acceptance of the condition on a daily basis (Westin et al., 2011). Recent thinking in psychology considers mindfulness in an even broader perspective than equanimity toward negative experiences (Lindsay and Creswell, 2015). It is suggested that a more flexible appraisal of oneself and others' attitudes translates from (and beyond) self-training, with a sustained influence on daily activities (ibid). This view addresses the issue of *persevering through difficulties*, with emphasis on the daily efforts required by patients with tinnitus. Among other approaches to psychotherapy, the attention that frustration has received in contemporary psychoanalysis (Bion, 2007; Ferro and Civitaresse, 2015) suggests that this approach might also contribute to our understanding of tinnitus tolerance from a psychological perspective.

## Limitations and strengths

The present study has two main limitations concerning data analysis and patients selection. Both involve the procedure of theoretical sampling, i.e., purposeful, category-driven choice of questions and participants in accordance with the Grounded Theory methodology (Draucker et al., 2007). First, the mode of recruitment (i.e., through the Internet) and the coding procedure in interviews did not sufficiently meet the requirements of theoretical sampling so this had a broader impact on data gathering. Purposeful sampling will be possible in the future, based on our understanding of a core category in tinnitus-induced disablement. However, we believe that our core category and supporting analysis demonstrate the relevance of the focused questions we used in the interview process. Second, our population cannot be considered as representative of the general tinnitus population. Participants were aging individuals who experienced long-term tinnitus, the shortest duration being 6 years at the time of the interviews. The testimonials gathered do not reflect the experience of a recent onset, nor the experience of younger participants. Likewise, problems related to work situations might have been underestimated since most of the participants do not work outside their homes. Furthermore, the study participants are members of the French tinnitus association. While the condition is a matter of concern for them, they strive to tolerate it in their daily life. Most of them, if not all, were disappointed as their expectations of finding therapeutic relief in encounters with health care professionals were not met. A different view on tinnitus and its implications for the individual patient might have resulted in a therapeutic approach more in line with what they expected. These limitations suggest the need for further studies using another set of interviews with purposeful selection of participants. Other types of patients (i.e., negative cases) will be sought such as those who do not report any variability of disablement, and others who are not disabled by tinnitus. There is also a need to consider participants' age (young adults), recency of onset and working status. The present study, however, offers an original, substantive theory of tinnitus-induced disablement and progressive tolerance. Predictable relationships are drawn from the present model that can be further tested by focused interviews, psychological investigations (see e.g., Kahn-Greene et al., 2006) or more recent neuroscience investigation of frustration (see e.g., Yu et al., 2014). Identifying a core category in the experience of tinnitus could lead to implementing new strategies to provide help in the various forms of tinnitus, from sustained suffering to reduced disablement through time. It could help in promoting meaningful clinical dialogue between patients and clinicians, which is essential for improving tinnitus tolerance.

## Conclusion

This study underlines the importance of variability in tinnitus-induced disablement when attempting to understand the condition in a long-term perspective. Furthermore, it highlights frustration as a central issue in chronic tinnitus. To our knowledge, this is the first study that clearly raises frustration to a conceptual level in the understanding of tinnitus-induced disablement, beyond anecdotal observations to date. Based on the results of the qualitative data analysis, we suggest that frustration influences the intra-individual variability of tinnitus annoyance. We further suggest that *modulating frustration* could be a new approach to tinnitus suffering, in contrast with current models of moderating *tinnitus interference* with individual perception. This perspective could have implications for counseling of tinnitus patients, e.g., by highlighting key variables in management such as focusing on the narrative of each individual. It also enlightens the recurrent difficulties in those who previously tolerated tinnitus, but who henceforth have to cope with frustrating events in their life (diseases, mourning, break-ups and so forth). Otherwise, the role of frustration in tinnitus-induced disablement may encapsulate the underlying rationale of person-centred counseling. Acknowledging the importance of tinnitus frustration invites practitioners to take into account the influence of the caregiver's attitude toward the condition. While many may consider that the lack of scientific explanation about the origin of tinnitus is the source of patients' disappointment, the participants in this study complained rather of the lack of dialogue with their practitioner. This means that primary care could start by paying attention to the suffering client and showing interest in his/her experience. We believe that the proposed model raises significant issues that can be incorporated into the clinical dialogue with the patient. The present findings may also have implications for basic research on non-auditory networks involved in tinnitus annoyance. There are many ways to try to ease individual frustration, and some of the current approaches, including counseling and psychotherapy, have a contribution to make. However, to our knowledge, it is still a matter of chance within hearing rehabilitation units whether or not patients with tinnitus receive the special care they need. Finally, this qualitative study will have achieved its goal if it results in future studies based on patients' testimonials, since patients' narratives have much to offer in improving tinnitus management from an individualized perspective.

## Author contributions

ND, SE, and RD designed the study. ND led the interviews with participants and collected data. Data analysis was performed by ND, SE, RD, and DA. ND, RD, and SE discussed the results. RD and SE provided critical revisions to the manuscript. All authors approved the final version of the manuscript for submission.

### Conflict of interest statement

The authors declare that the research was conducted in the absence of any commercial or financial relationships that could be construed as a potential conflict of interest.
